# A Direct Attack on the Tubercle Bacillus

**Published:** 1900-12-29

**Authors:** 


### A DIRECT ATTACK ON THE TUBERCLE BACILLUS.

In the last of the three Harveian Lectures, Dr. Maguire propounded a mode of treatment which he has devised, the object of which is (1) to hinder the action of the bacillus tuberculosis by destroying its congeners, the staphylococcus pyogenes and the pneumococcus; (2) to hinder its action by interfering with its own vitality; and (3) possibly to kill it *in situ*. The proceeding may be described as an attempt to restore the asepsis of the lung. Dr. Maguire says that inhalations cannot be of sufficient strength to effect this; subcutaneous injections are so diluted as to be rendered powerless before they reach the lung; and direct injections into the lung cannot reach every spot which is infected. But, he says, anything injected into the peripheral veins must necessarily first pass to the lungs, diluted only with the contents of the right ventricle at the time of its arrival at that cavity. After many experiments, which he described, he determined to try the effect of antiseptics alone, and he selected formic aldehyde as the one most likely to be of use. Using first a strength of 1 in 250,000 he mounted up to a solution of 1 in 2,000, and found that he could inject 30 minims, or 2 c.cm., in the space of five heart-beats; which would mean that for five heart-beats the lung would be washed out with a solution of formic aldehyde having a strength of 1 in 500,000. Further experiments, however, have made him hope that he will be able to get a sluicing of the lung, during a considerable number of heart-beats, with a solution of 1 in about 50,000. Some of this solution will remain in the lungs, and another portion will be brought back again to the lungs after having been passing through the general circulation, so that the total effect on the lung tissue will be greater than that is represented by the immediate effect of the injections. The cases submitted to this treatment were all such as showed pronounced lesions. About 70 such cases had been treated. Nearly all showed some improvement, and some have demonstrated absolute disappearance of tubercle bacilli from the sputum. We hope that Dr. Maguire will continue his researches. To directly get at the bacilli has been the desire of many experimenters, but no one, we fancy, has yet got so near his goal as Dr. Maguire has done. It must be obvious, moreover, that if he can succeed in this attempt he will have gained a point of vantage from which many other diseases may perchance be attacked. But all this work is a case of using double-edged weapons, and while we urge Dr. Maguire to continue, we recognise that in less skilled and less careful hands this "sluicing" of the pulmonary tissues with antiseptics would be a dangerous game, and it is much to be hoped that the suggested method will be left for the present to skilled experimenters, and will not be dabbled in by tyros until all the necessary details have been worked out—which is not yet.

